# Wear and Dynamic Mechanical Analysis (DMA) of Samples Produced via Fused Deposition Modelling (FDM) 3D Printing Method

**DOI:** 10.3390/polym16213018

**Published:** 2024-10-28

**Authors:** Jiri Struz, Miroslav Trochta, Lukas Hruzik, Daniel Pistacek, Sylwester Stawarz, Wojciech Kucharczyk, Miroslaw Rucki

**Affiliations:** 1 Faculty of Mechanical Engineering, VSB—Technical University of Ostrava, 17. Listopadu 2172/15, 70800 Ostrava, Czech Republic; miroslav.trochta@vsb.cz (M.T.); lukas.hruzik@vsb.cz (L.H.);; 2 Faculty of Mechanical Engineering, Casimir Pulaski Radom University, Stasiecki Str. 54, 26-600 Radom, Poland; stawarz@urad.edu.pl (S.S.); wojciech.kucharczyk@uthrad.pl (W.K.); 3 Institute of Mechanical Science, Vilnius Gediminas Technical University, J. Basanaviciaus Str. 28, LT-03224 Vilnius, Lithuania; miroslaw.rucki@vilniustech.lt

**Keywords:** FDM, coefficient of friction, weight loss, DMA, storage modulus, loss modulus, glass transition temperature

## Abstract

In recent years, plastic and metal 3D printing has experienced massive development in the professional and hobby spheres, especially for rapid prototyping, reverse engineering, maintenance and quick repairs. However, this technology is limited by a number of factors, with the most common being the cost and availability of the technology but also the lack of information on material properties. This study focuses on investigating the material properties of PLA, PETG, HIPS, PA, ABS and ASA in order to elucidate their behavior in terms of wear and thermal resistance. The research builds on previous studies focusing on the mechanical properties of these materials and includes wear testing and DMA analysis. Weight loss, frictional forces, and frictional work including relative frictional work are recorded as part of this testing. The storage modulus and loss modulus including tan(δ) were then measured using DMA.

## 1. Introduction

In recent years, there have been very rapid developments in the fields of 3D scanning, reverse engineering, rapid prototyping, artificial intelligence and, last but not least, additive technologies. To a large extent, modern methods and approaches such as reverse engineering, rapid prototyping, etc., are based on advances in additive technologies, more specifically 3D printing. It is a modern method that has experienced and is still experiencing very rapid development in the last decade. Thanks to this method, it is possible to achieve very interesting products in terms of design, and, on the other hand, it allows designers to significantly speed up the design process. As it happens, areas that develop rapidly create a so-called scientific or research gap [1], and many aspects of this technology are still unexplored. Since it is possible to print products from both plastics and metals, and there are a variety of 3D printing methods such as FDM (fusion deposition modeling), SLA (stereolithography), SLS (selective laser sintering), etc., this “scientific gap” is still wide. The FDM 3D printing method is very popular in both the professional and hobby communities [2]. A limiting factor when used in a professional environment may be the lack of information on certain parameters. One group of such information may be information on the friction and wear of FDM products.

There are a number of papers on this issue, which are always limited to some part of the issue. In general, research teams often deal with PLA (polylactic acid) or ABS (Acrylonitrile Butadiene Styrene) plastics. The central topic is usually how printing parameters affect the friction and wear of the samples, or how to optimize these parameters [3,4,5,6,7,8,9,10,11]. The research results [4,5,6,9,11,12,13] agree that printing parameters, such as layer thickness, pattern and fill density, are key factors affecting the mechanical properties, wear and coefficient of friction (COF). The studies of [5,9,11] agree that the greater the layer thickness, the less the wear, and [11] also states that there is a decrease in the coefficient of friction for PLA-PCU (PCU—polycarbonate urethane). In the case of PA (polyamide) material, according to [12], as the layer thickness increases, the friction coefficient increases while the mechanical properties decrease. Changing the nozzle temperature from 240 °C to 260 °C causes an increase in mechanical properties, but the coefficient of friction does not change significantly. During polishing, significantly higher friction coefficients were measured due to the change in surface structure. In the research reported in [9], it was shown that wear decreases with increasing layer thickness and nozzle temperature but increases with higher printing speed. For ABS material, [6] provides a prediction equation based on statistical methods to predict wear. The results of [13] suggest that the orientation during printing has an effect on the COF and wear depth, with the best results obtained when printing horizontally. According to [14], variations in the coefficient of friction (COF) may be caused by differences in the fiber composition of the materials. It is in this study that two flexible polymers TPU92 (thermoplastic polyurethane, TPU-based polymer of Sava Filament©, Bayramiç, Turkey) and CARBOV60 (TPU-based polymer of Sava Filament©, Bayramiç, Turkey) are compared for which differences in the COF are evident. Publication [5] compares ABS and CFPLA (PLA with 20% carbon fiber) materials, while [7,15], on the other hand, discuss the influence of the graphite content in ABS material in terms of tribological properties. The research in [5] demonstrates that CFPLA material has a significantly higher wear resistance compared to ABS material, due to just the use of carbon fibers that promote strength, stiffness and hardness. The results also show that the wear strength, specific wear rate and friction coefficient of CFPLA are lower for lower layer thickness, high-density filler and grid pattern. ABS composites filled with graphite powder [7] or the addition of graphite to the PLA top layer [15] leads to a reduction in the coefficient of friction and wear of the printed parts. Similar conclusions are also reached in [8], where nano-aluminum-reinforced PLA exhibits lower friction and wear coefficients, with both wear losses and friction values decreasing with increasing nano-aluminum mass fraction in the PLA matrix. For PA6 material, the glass- and carbon-fiber-reinforced samples showed better properties in terms of hardness and durability after heat treatment. The results also show that the glass-fiber-reinforced samples exhibit a lower coefficient of friction and lower volume loss than the carbon-fiber-reinforced samples. On the other hand, [16] deals with the orientation of carbon fibers in the PEEK (polyether ether ketone) polymer matrix. This shows that the orientation of carbon fibers significantly affects the tribological properties of composites produced by FDM. Lower wear was found for tilted fiber orientation compared to parallel or antiparallel orientation. The inclined orientation also exhibited the best behavior under microscratching, except for the lowest normal load. Investigations were also performed on PLA/Cu filament [17], where the best results were obtained at a 230 °C nozzle temperature, 70 °C plate temperature and 0.14 mm layer height. Testing of the filamentary composites was carried out in [18], where the effect of an innovative head with symmetric matrix feeding was investigated. At the same time, significant differences in the friction process in dry and wet environments were observed. The fibrous composites exhibited ten-times-lower wear intensity and two-times-lower friction values in water than in air, with more uniform surface wear in the aqueous environment. The results of [19] suggest that conventional friction models (Amontons–Coulomb, Tabor–Bowden, and variants of the Hertz contact model) may be suitable for describing friction on FDM surfaces if properly calibrated based on empirical data.

The above analysis, which is supported by scientific publications and facts based on the references listed in the last section of this study, shows that research in the field of friction, tribological properties and mechanical properties in 3D printing is serious and necessary. This is particularly important in the context of the future use and development of this field for the needs of industrial practice as well as for the lay and scientific public. Increased knowledge of this issue can bring desirable economic as well as environmental savings and benefits. It is for this reason that it is essential to better describe and complement the state of knowledge in this area. Current research often focuses on materials such as PLA and ABS, but there is a need to broaden the focus to the full range of the most commonly used commercial materials, such as PETG (polyethylene terephthalate glycol), ASA (acrylonitrile styrene acrylate), PA (polyamide), HIPS (high-impact polystyrene) and others. In this way, a better understanding and optimization of the tribological properties and mechanical characteristics of these materials can be achieved, leading to more efficient use in various applications.

The search also found articles dealing with the interaction of steel and plastic material produced by 3D printing, as well as a comparison of a turned plastic specimen. Article [20] presents a comparative study of the frictional properties between PETG, PLA with aluminum, ABS, TPU and C45 alloy steel, which is often used in industrial applications for its excellent mechanical strength and durability. Paper [21] compares the frictional properties between a turned ABS specimen with a 3D-printed specimen. The paper shows that the dynamic coefficient of friction is higher for the turned ABS sample, which implies greater instability in its frictional behavior. By comparing the average wear values of each specimen during the measurements, it was found that the wear level of 3D-printed ABS is lower than that of the turned ABS.

Dynamic mechanical analysis (DMA) is another parameter that determines the usability of the material in practice. These analyses can provide basic data on mechanical properties that are directly related to the processing and workability of the material. DMA is considered to be one of the most accurate methods to determine the glass transition and other transitions in polymers. This method can be used to determine the glass transition temperature, softening and melting points, mechanical losses in the material—damping capacity, gradual change in dimensions of materials under constant load, degree of crystallization, thermal stability, etc. [22]. The available literature shows that this issue in the field of plastics for 3D printing is a topic that needs to be addressed. One of the studies dealing with ABS and PLA materials is [23]. The data obtained show that ABS shows good energy absorption capabilities up to 90 °C, while PLA up to 48 °C, with ABS releasing energy in the form of heat from 110 °C onwards. In contrast, PLA has this capability up to 58 °C. Study [24] compares PLA material samples that were plasma treated after 3D printing, before printing and without treatment. Due to these treatments, decreases in the glass transition temperature were observed. The plasma-treated samples exhibited a higher storage modulus as well as higher elasticity. In [25], samples of PETG material produced using the FFF (Fused Filament Fabrication) 3D-printing method were analyzed. PETG is a variant of PET known as PETG—polyethylene terephthalate glycol. Compared to PET, PETG is a tougher material with higher impact strength. PETG also has better chemical resistance and is easier to process. This is why it has found a place in 3D printing, for example [26]. The effects of the changes on the viscoelastic properties were investigated. The samples showed a decrease in storage modulus with increasing temperature, with the loss modulus increasing up to the glass transition temperature. Maximum values of storage modulus were achieved using a layer height of 0.17 mm, regardless of frequency, while in the case of loss modulus, maximum values were recorded at a layer thickness of 0.3 mm. Investigation of the PETG material samples in the study reported in [25] found that frequency has a minimal effect on the storage modulus but a significant effect on the loss modulus. Research [27] focuses on recycled PET (polyethylene terephthalate) with shape memory. For these materials, an increase in storage modulus was measured during heating prior to the glass transition temperature range. A special case may be the eco-friendly materials addressed in [28]. It was found that the storage modulus decreases with test temperature (range 30 °C to 120 °C) as well.

The previous paragraph shows that DMA testing has only been carried out for certain types of materials, such as PLA, ABS and PETG. However, there is a lack of a comprehensive study of a larger number of materials and how they compare to each other, which would allow engineers and hobby users to better use the knowledge and information gained and use 3D-printed products more frequently in their applications.

## 2. Materials and Methods

In view of the very frequent use of the FDM 3D-printing method by the general public, but also by professionals, we decided to use the Prusa i3 MK3S 3D printer (©Prusa Research Plc., Prague, Czech Republic) with cover for sample preparation. The materials used as sample materials were ASA, ABS-T, PA, PLA, PETG, HIPS. These materials were chosen with respect to their properties, because according to [29,30,31], ABS material can be considered very versatile, showing higher strength than objects made of PLA material. Compared to, e.g., ABS material, PLA stands out for its simplicity in printing and it is biodegradable. It is relatively easy to add other materials to its composition and, thus, PLA-based materials with particles of wood, steel, bronze, etc., can be created. A compromise between these materials can be PETG. PETG material represents the most commonly used material at the present time. The advantage of the material is that it is recyclable and food safe. This material should be durable but not biodegradable. PA material was chosen for its durability, high flexibility and its general use in industrial applications. HIPS is also one of the most widely used materials, particularly for its strength, toughness and good temperature stability. ASA is very similar to ABS, characterized by UV resistance, mechanical strength, rigidity and temperature resistance. These samples were subjected to tribological testing on the TT-4 Tribotester described in research [32], which was designed and manufactured at the Casimir Pulaski Radom University and DMA testing on the DMA/SDTA861e (Mettler-Toledo GmbH, Schwerzenbach, Switzerland).

### 2.1. Sample Preparation

**Figure 1:**
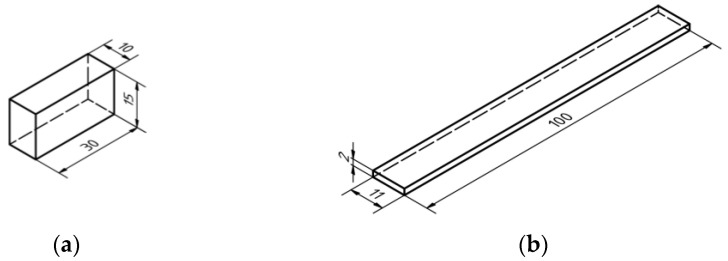
Samples dimensions—(**a**) sample dimensions according to Table 1, (**b**) sample dimensions according to Table 2.

As part of this testing, samples were designed for tribological parameter testing using the TT-4 Tribotester as well as samples for DMA testing. The geometry of the samples is outlined in Figure 1. The sample geometry respects the experience and design capabilities of the TT-4 Tribotester and DMA test device. Table 1 summarizes the basic parameters about the samples used for wear testing. In the case of printing temperatures, the recommendations of the material manufacturers were followed.

**Figure 2:**
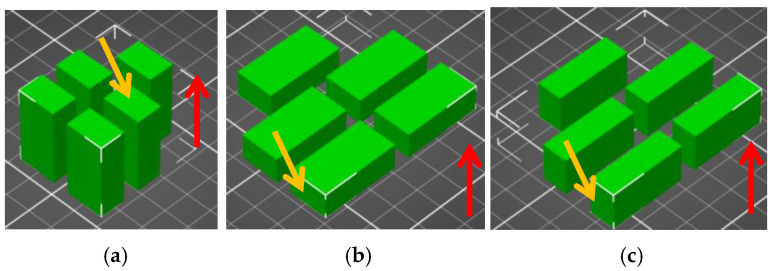
Orientation of samples for wear testing in PrusaSlicer—(**a**) sample group designated XX-100-030-1, (**b**) sample group designated XX-100-030-2, (**c**) sample group designated XX-100-030-3 (the letters XX are replaced by the material designation in Table 1, e.g., PLA).

Fifteen samples of these materials were printed under these conditions, always in variations of five samples with different orientations to the pad. A straightforward filling was used, which was always angled at 45° to the longitudinal direction, and each layer held a 90° angle between itself. The orientation, printing direction (red arrow) of the samples and test surface (orange arrow) are shown in the images directly from the PrusaSlicer program, see Figure 2. The labeling of the samples shown in Figure 2a corresponds to XX-100-030-1, the samples in Figure 2b correspond to XX-100-030-2, and the samples labeled XX-100-030-3 correspond to Figure 2c. The letters XX represent the sample material.

In the case of DMA testing, the samples were printed under the conditions described in Table 2. Five samples were printed for each material. The fill settings were the same as for the wear test specimens.

**Figure 3:**
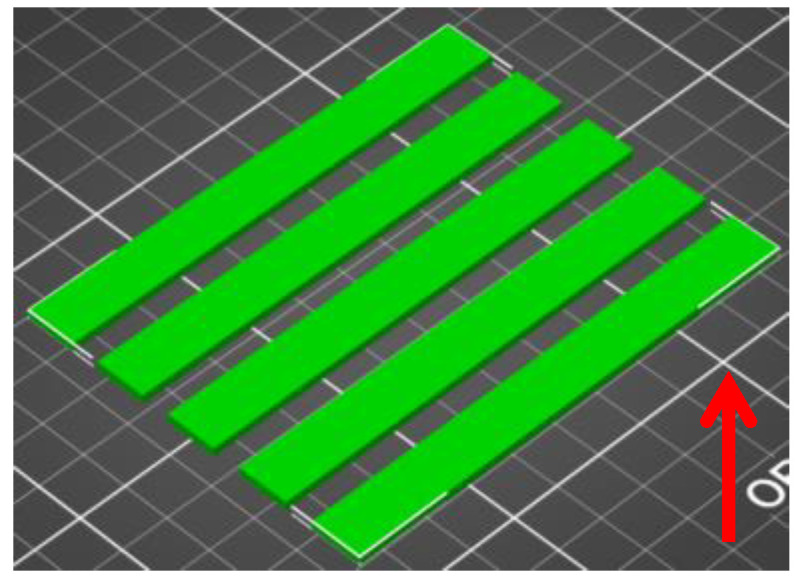
Orientation of samples for DMA test in PrusaSlicer.

As in the previous case, the orientation and print direction (red arrow) of the samples are shown in Figure 3.

### 2.2. Wear Measurements

**Figure 4:**
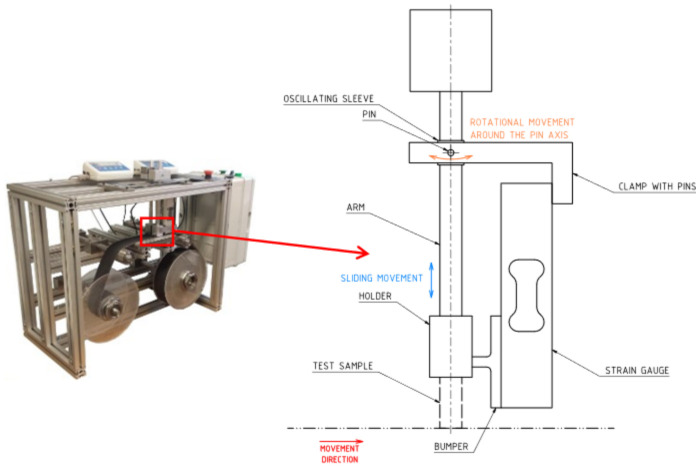
Wear measuring equipment—TT-4 Tribotester [32].

The durability of a material is often related to its resistance to wear. This resistance depends mainly on its composition and manufacturing processes [33]. In testing, the wear resistance can be determined by measuring the material loss over time while recording the load and surface velocity values [34]. By recording these parameters, the wear coefficient can be determined. As these parameters are individual, the wear coefficient is a relative quantity, and it is always necessary to ensure that these conditions are the same if materials are to be compared. It is for this reason that the TT-4 device, a schematic of which is shown in Figure 4, was used for the wear tests.

The principle of operation of this device is based on the sliding of the sample on a friction belt moving at a certain speed. A normal force generated by a weight is applied to the specimen and a frictional force is induced between the specimen and the friction belt, which is recorded by an extensometer. The design of the equipment is such that the results are not significantly affected by vibration and temperature rises. As a result, the ranges and standard deviations are then significantly smaller than when using pin-on-disc devices [32]. In order to perform friction tests, material samples of fixed dimensions were produced to allow their installation in the TT-4 tester, see Figure 2. Each time, a new sample and a new fragment of friction tape were used. Table 3 summarizes the conditions during the test. Figure 4 also shows the measuring head in more detail. One of the most important elements of the measuring head is the oscillating sleeve. The sleeve is attached to the clamp with pins. Fixing the sleeve in the buckle with pins allows it to move around the pin axis. The sample is abraded during the test, which causes its length to change. Manufacturing the sleeve with appropriate dimensional tolerances allows the arm to slide freely in it in the up–down direction. This solution allows you to avoid changes in the arm length during the test caused by changes in the sample length. The tested sample is mounted in the holder. Due to the operation of the friction band, the sample exerts pressure on the strain gauge through the bumper.

From the results obtained for each sample, the average friction force *F_fa_* (N) was calculated. To avoid gross errors in the initial and final stages, the first two and last two measurement results were excluded from further processing. Next, the frictional work *W_f_* was calculated using Equation (1):

(1) $$W_{f}=F_{fa}\times s_{d}$$

where *s_d_* represents the sliding distance.

Durability was measured as the specific work of wear *We_f_* given by Equation (2), in which the friction work *W_f_* and the mass lost during the test are Δ*m*. Material loss was measured on a Radwag 0.01/0.1 mg Modello AS82/220.R2 (Radwag, Radom, Poland) with a precision of 0.01 mg.

(2) $$W_{ef}=\frac{W_{f}}{\Delta m}$$

**Figure 5:**
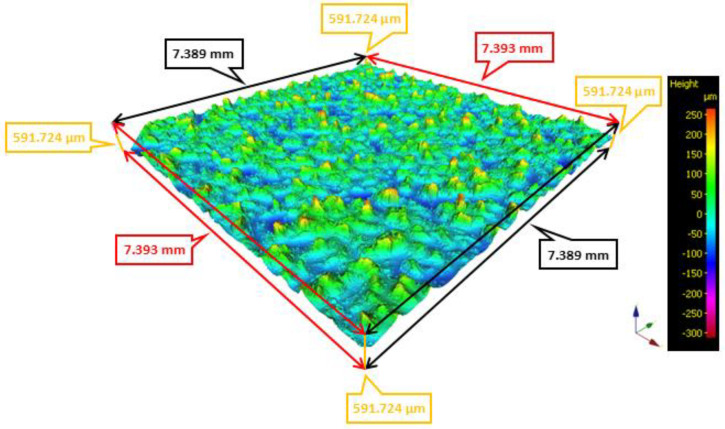
Surface structure of the friction belt.

The friction belt sample was measured on an Alicona InfiniteFocus (Alicona Imaging GmbH, Graz, Austria) optical 3D metrology machine. The surface structure of the friction belt is shown in Figure 5. The device setup during testing is summarized in Table 4. Table 5 then summarizes the S-parameters and V-parameters of the friction belt surface.

### 2.3. DMA Test

**Figure 6:**
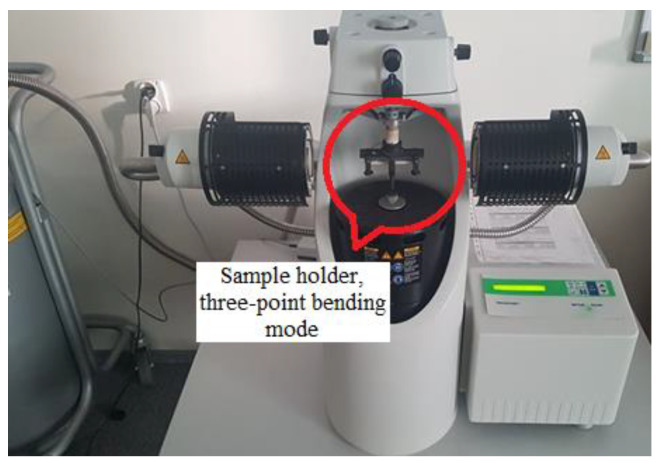
DMA test device.

The dynamic device DMA/SDTA861e (manufactured by Mettler-Toledo GmbH, Greifensee, Switzerland) was also used in this research. The device is described more precisely in [35,36] and is illustrated in Figure 6.

The advantage of this device is that we were able to obtain information about the materials tested, also including the following:

- Young’s modulus, the modulus of longitudinal elasticity;
- Kirchhoff’s modulus, which means the shear modulus;
- Plastic deformation and stress relaxation characteristics;
- Damping characteristics and data on viscoelastic behavior.

As can probably already be seen from the shapes of the test specimens for DMA testing, the testing is based on a three-point bend. The parameters and test conditions for DMA testing are shown in Table 6 for each material and sample separately.

## 3. Results

This section summarizes the results of the wear measurements on the TT-4 Tribotester and also presents the results of the DMA test device measurements.

### 3.1. Results from Wear Measurements

**Figure 7:**
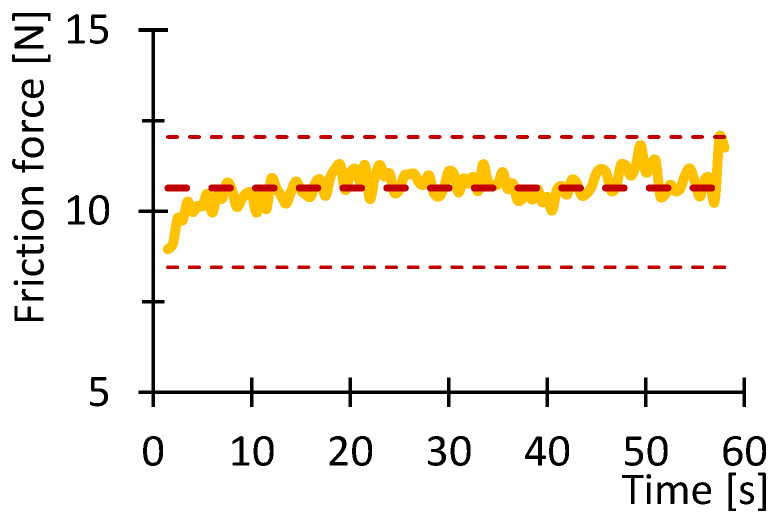
Recorded friction force as a function of time for the PLA_100_030 sample.

**Figure 8:**
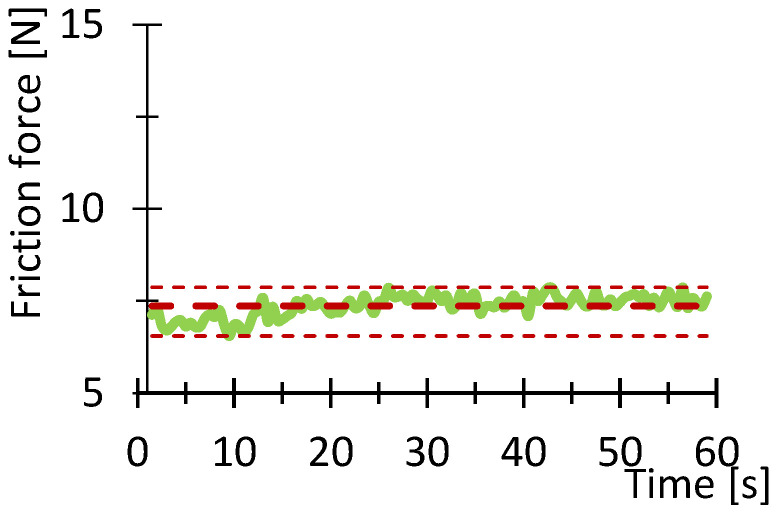
Recorded friction force as a function of time for the PETG_100_030 sample.

**Figure 9:**
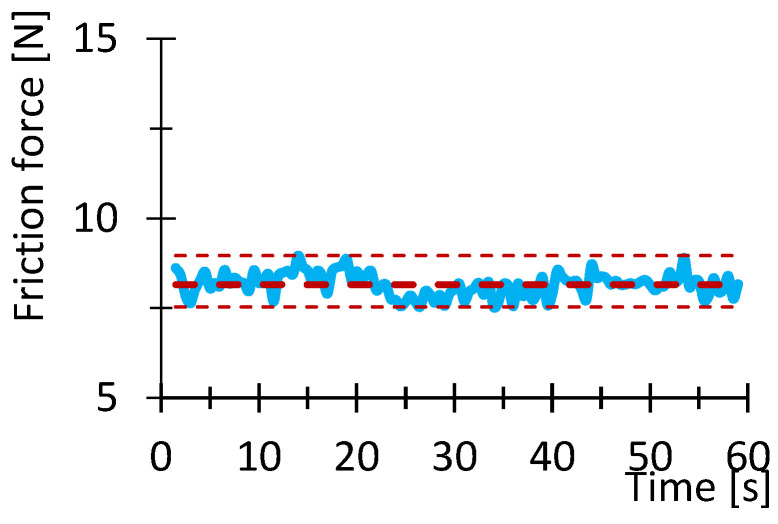
Recorded friction force as a function of time for the HIPS_100_030 sample.

**Figure 10:**
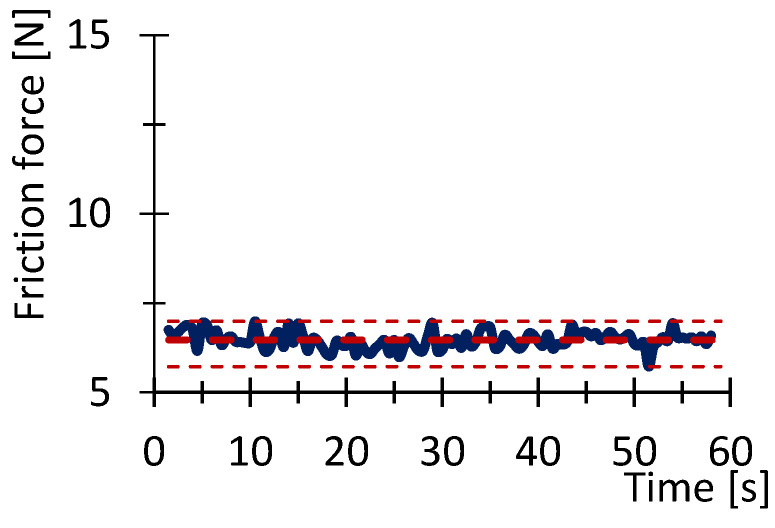
Recorded friction force as a function of time for sample PA_100_030.

**Figure 11:**
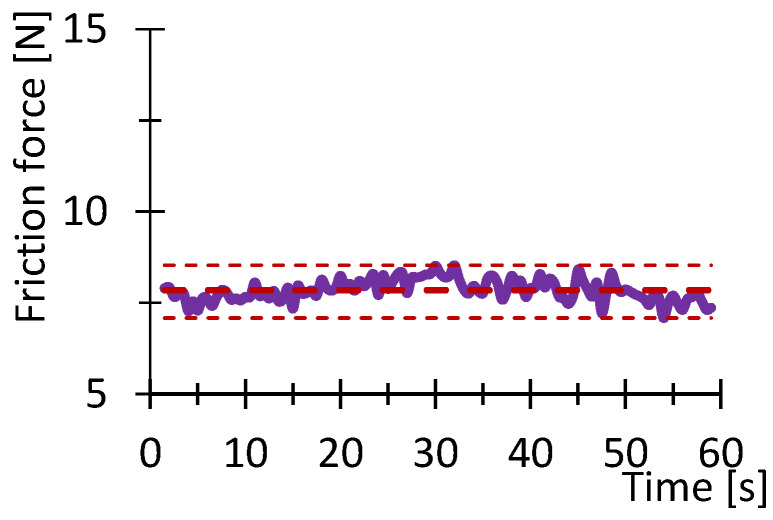
Recorded friction force as a function of time for the ABS_100_030 sample.

**Figure 12:**
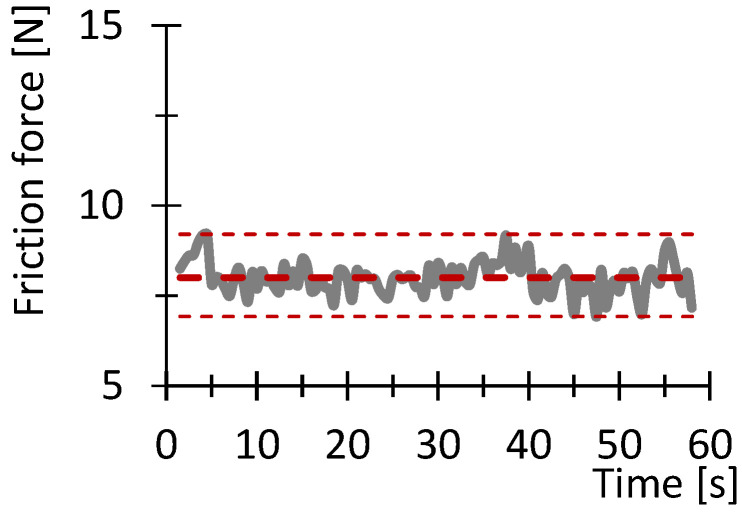
Recorded friction force as a function of time for the ASA_100_030 sample.

**Figure 13:**
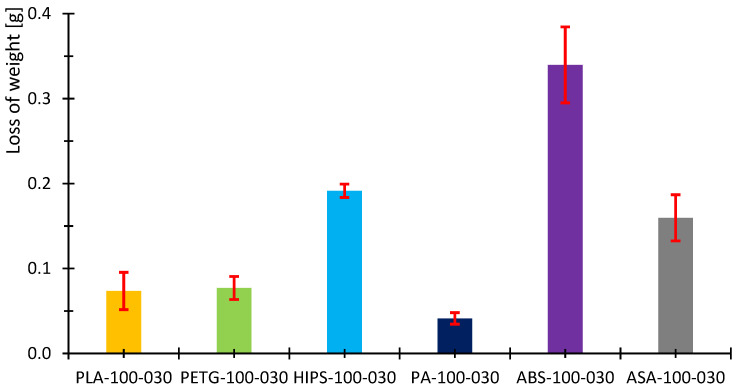
Calculated average mass loss for the tested samples.

**Figure 14:**
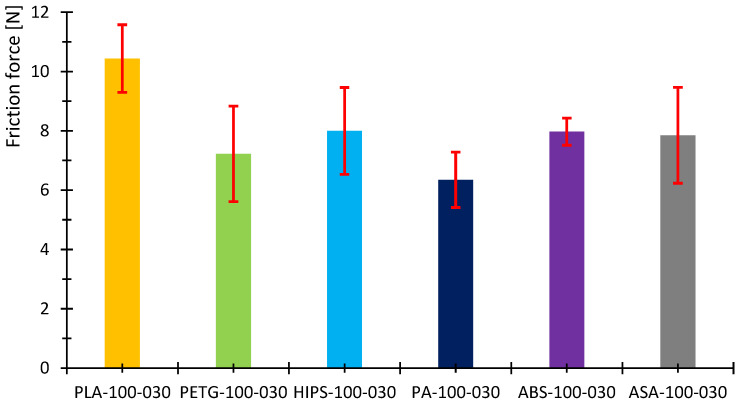
Calculated average friction force for the tested samples.

**Figure 15:**
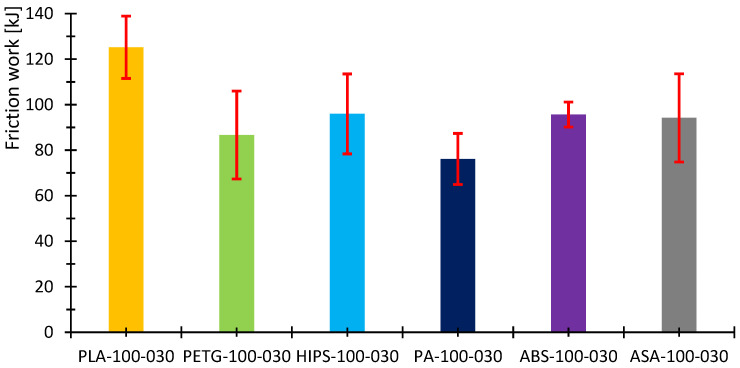
Calculated average friction work for the tested samples.

Figure 7, Figure 8, Figure 9, Figure 10, Figure 11 and Figure 12 show graphs, in which the average force versus time is recorded. In the graphs, the mean value is also indicated by a thicker dashed line; the measured maximum and minimum values are indicated by thin dashed lines. The average values of the weight loss of the material sample without context to the original weight (Figure 13) and the average value of the friction force (Figure 14) including the average friction work (Figure 15) were then evaluated on the basis of these data.

The measured data show that the highest frictional force (10.3 N) and frictional work (123.3 kJ) were performed by the PLA sample. On the other hand, the lowest value of friction force and, therefore, frictional work can be observed for the PA material, including the lowest weight loss. The highest material loss, over 0.3 g, was observed for ABS material, which exhibited a friction force of 8 N and a frictional work of 95.7 kJ. In terms of frictional force and work, PETG material appears very similar to ABS material, as the frictional force and work were 7.2 N and 86.7 kJ, respectively. The HIPS and ASA materials also behaved very similarly in terms of this test. The magnitude of the friction force reaches values of 8 N and 8.8 N, respectively. The magnitude of the frictional work is 95.9 kJ and 105.3 kJ, respectively. The magnitude of the mass loss is 0.19 g and 0.18 g for the HIPS and ASA materials. The measured data show that the lowest frictional force was achieved for PETG and PA, while the material loss reached 0.1 g. On the other hand, the highest friction force was achieved for PLA materials with still satisfactory magnitude of material loss (up to 0.1 g).

Table 7 briefly summarizes the measured values. A colored circle with a pipe or cross indicates the best or worst result. The other green circles represent weight loss up to 0.1 g, while the orange color represents the result between the green results and the worst result. In the case of frictional force and work, the marking is the same. The green circle with a pipe represents the lowest result, while the red circle with a cross represents the worst result. The green circle then represents a result very close to the best value and the orange one between this value and the worst result. The measured data also show that the standard error in the measurement of weight loss is highest in the case of ABS and PLA but also ASA. The highest stability of the results was achieved for HIPS and PA. On the other hand, in terms of frictional force and frictional work, the largest variance in values was observed for PETG, HIPS and ASA materials. The lowest ripple values were obtained for PLA, PA and ABS. Table 8 summarizes the results of the specific frictional work.

### 3.2. DMA Test Results

**Figure 16:**
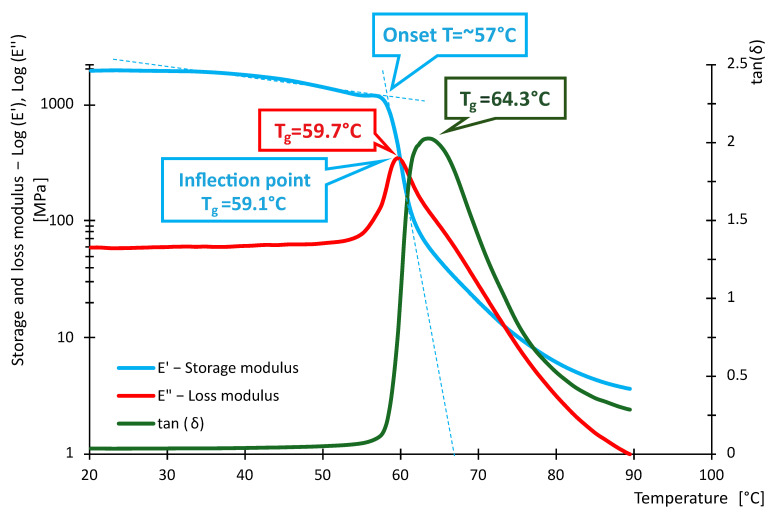
Storage and loss modulus of PLA-100-030-DMA samples.

**Figure 17:**
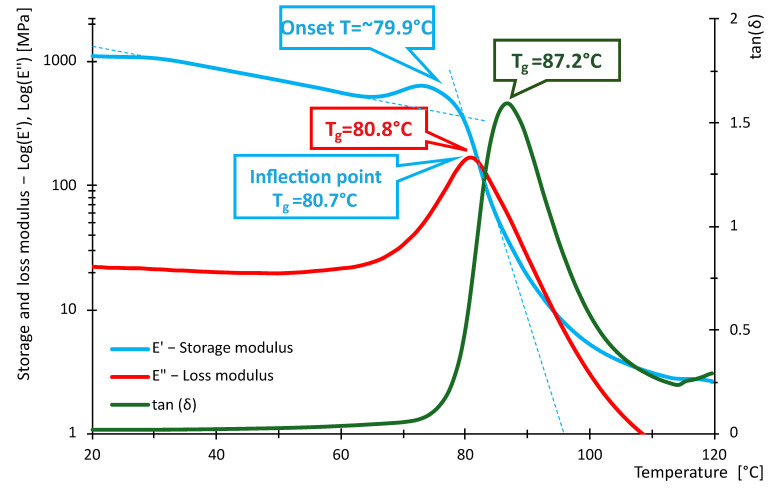
Storage and loss modulus of PETG-100-030-DMA samples.

**Figure 18:**
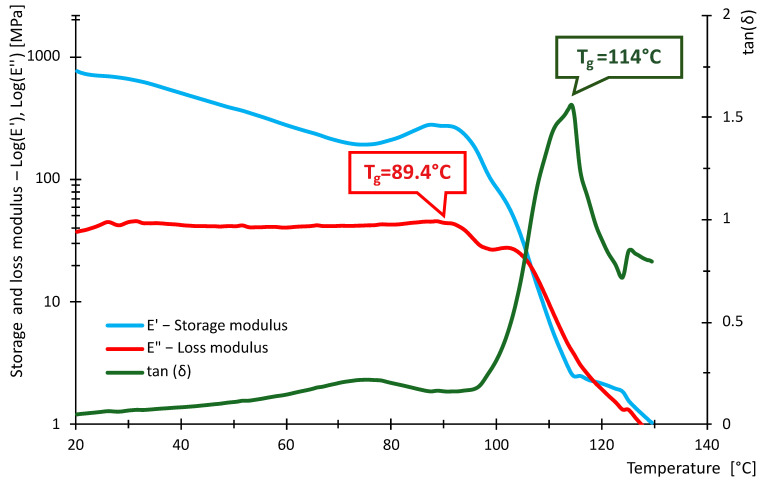
Storage and loss modulus of HIPS-100-030-DMA samples.

**Figure 19:**
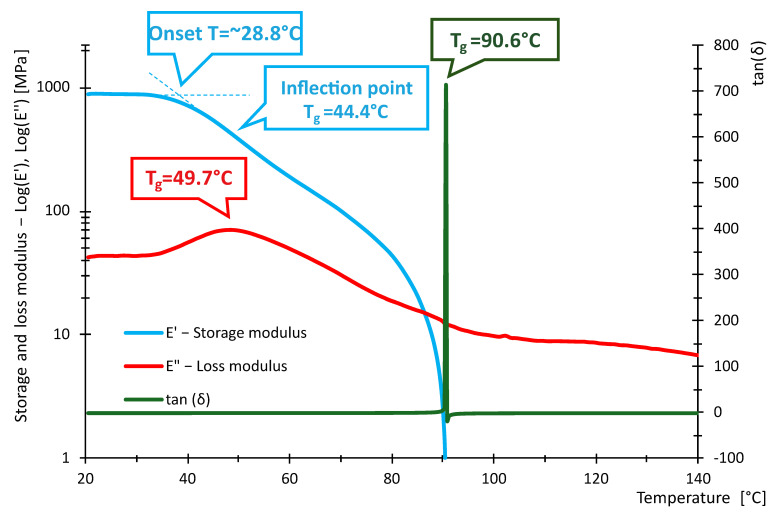
Storage and loss modulus of PA-100-030-DMA samples.

**Figure 20:**
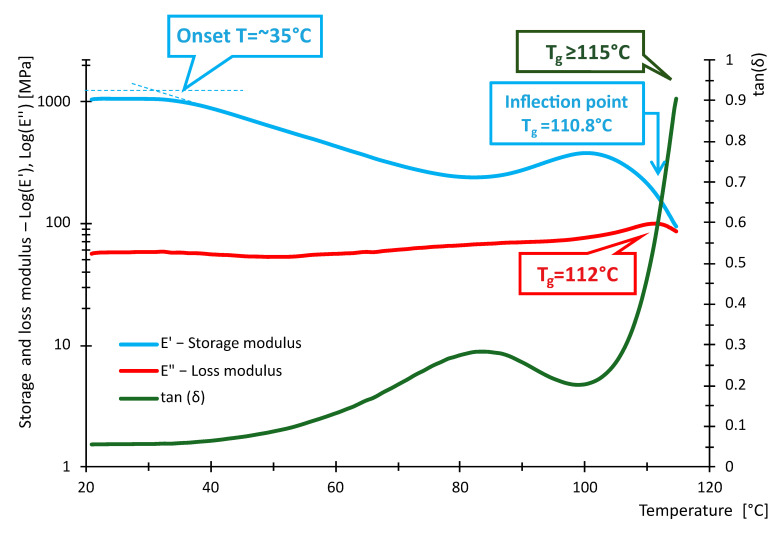
Storage and loss modulus of ABS-100-030-DMA samples.

**Figure 21:**
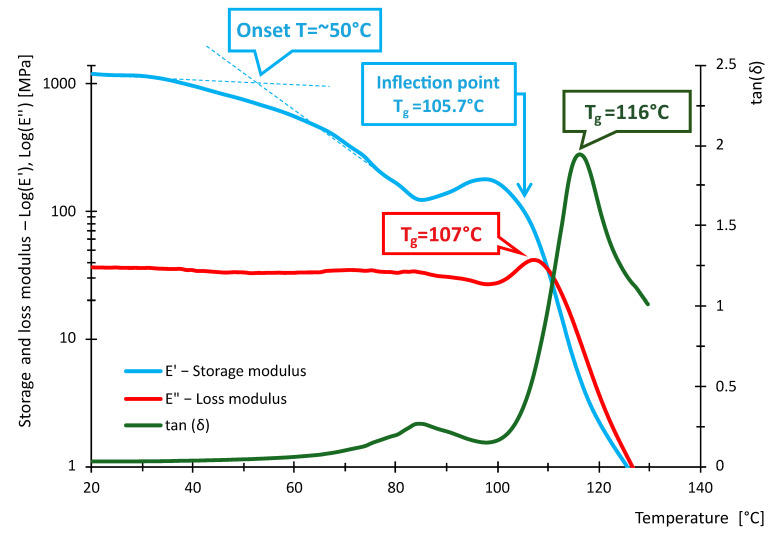
Storage and loss modulus of ASA-100-030-DMA sample.

The following Figure 16, Figure 17, Figure 18, Figure 19, Figure 20 and Figure 21 show the measured values of storage modulus and loss modulus including *tan*(*δ*) for each material tested.

For the PLA material, we measured values of *T_gδ_* = 64.3 °C, *T_gE″_* = 59.7 °C and *T_gE_* = 59.1 °C. These results indicate that PLA has a glass transition temperature ranging from 59.1 °C to 64.3 °C. For PETG material, *T_gδ_* = 87.2 °C, *T_gE″_* = 80.8 °C and *T_gE′_* = 80.7 °C were measured. Thus, PETG exhibits higher temperature than PLA, indicating that PETG is more temperature resistant and more suitable for applications requiring higher thermal stability. In contrast, the HIPS material showed *T_gδ_* = 114 °C, *T_gE″_* = 89.4 °C, while *T_gE′_* could not be evaluated. In the case of PA, the experiment showed values of *T_gδ_* = 90.6 °C, *T_gE″_* = 49.7 °C and *T_gE′_* = 44.4 °C, showing significant differences in glass transition temperatures. For ABS material, we measured *T_gδ_* higher than 115 °C, *T_gE″_* = 112 °C and *T_gE′_* = 110.8 °C. Thus, ABS exhibits very high thermal resistance with glass transition temperatures around 110.8 °C to 115 °C, making it suitable for use in applications where the material is exposed to higher temperatures. In contrast, *T_gδ_ =* 116 °C, *T_gE″_* = 107 °C and *T_gE′_* = 105.7 °C were measured for the ASA material. ASA exhibits high thermal resistance with glass transition temperatures between 105.7 °C and 116 °C. These values are comparable to ABS, making ASA a suitable material for applications requiring high temperature and weather resistance.

In terms of damping capacity, more or less constant values can be observed for PLA material up to approximately 40 °C, with a gradual increase at this temperature. The increase is not noticeable in the region between 50 °C and 60 °C. The situation for PETG material is quite similar, where *tan*(*δ*) values are constant up to about 45 °C. A significant increase can be observed between 70 °C and 90 °C. The PA material shows constant values up to 40 °C. At this temperature, a gradual increase begins, where a steep increase in *tan*(*δ*) values and, thus, damping capacity can be observed, especially between 80 °C and 90 °C. The HIPS material shows a gradual increase in the damping effect, where a slight decrease can be observed between 80 °C and 100 °C, followed by a rapid increase around 100 °C. The ASA material behaves quite similarly in this respect, with a constant to slightly increasing *tan*(*δ*) values up to 60 °C. A steeper rise and fall can be observed around 85 °C and a sharp rise around 102 °C. For the ABS material, a gradual increase from about 40 °C with a slight decrease followed by a significant increase between 80 °C and 110 °C was observed. Table 9 summarizes the measured values between 20 °C and 40 °C. The purpose of Table 9 is to summarize the properties of individual materials under fairly standard ambient conditions when the temperature does not rise above 40 °C.

Based on the measured values of the glass transition temperatures *T_g_*, it can be concluded that the tested materials show different thermal resistance. PLA appears to be the least-temperature-resistant material, while ABS and ASA show the highest temperature resistance, making them suitable for demanding applications. PETG and HIPS also show good thermal properties. These results can serve as a basis for selecting the appropriate material for specific applications, where heat resistance is key.

The characteristics of the damage and permanent deformation of the individual samples are shown in Table 10.

**Figure 22:**
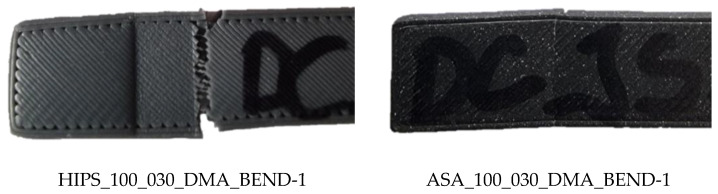
Detail of incomplete damage in HIPS and ASA samples.

Table 10 shows the shape and condition of the samples after the test. For the PLA samples, it can be noticed that there is no significant physical damage, but the shape of the sample is significantly bent, see side view. On the other hand, the PETG material is also not significantly physically damaged. Compared to the PLA material, it can be seen that the PETG material shows a wavy shape in the side view. Physical damage was also not detected in the samples of the PA material; the sample shows a slight waviness, very similar to the samples of the HIPS material. Physical damage during the test also did not occur in the samples of the ABS material, whereas it did in the samples of the ASA material. Both materials show a very low waviness of shape in lateral view. This shape can be considered more or less planar. In the case of the HIPS material, physical damage occurs at the location of the three-point bending support on both sides. On the one hand, the materials were completely separated from each other; on the other hand, damage can be seen with the naked eye, but complete separation did not occur. A similar pattern of damage can be observed in the ASA material samples, see Figure 22. The HIPS material sample shows some waviness, which does not reach the same intensity as in the case of PETG material.

The indentations of the three-point bending supports are present on all the specimens examined, with the ABS, PETG and HIPS specimens being the most pronounced. Both completed and non-completed physical damage to the ASA and HIPS specimens occurs near the three-point bending supports on the inside of the specimen.

## 4. Discussion

Table 11 summarizes the measured values of the glass transition temperature, including a comparison with commonly reported tabulated values for filaments or plastic materials outside of 3D printing.

From the data in Table 11, it can be seen that the measured values of the glass transition have either little or no deviation from the tabulated values given by the manufacturers. With respect to the fact that these are materials intended for 3D printing, the *E*′ and temperature dependencies are similar to the general waveforms of amorphous or semi-crystalline materials. The measured data show that PLA and PA exhibit the lowest temperature resistance, while ASA and ABS exhibit the highest temperature resistance. The curves shown in Figure 16, Figure 17, Figure 18, Figure 19, Figure 20 and Figure 21 show that the so-called onset temperatures when the storage modulus *E*′ starts to decrease are, for PLA, approx. 57 °C; PETG, approx. 80 °C; PA, approx. 30 °C; ABS, approx. 35 °C; ASA, approx. 50 °C.

If the results of DMA testing and wear work are related to each other, it can be noticed that the PLA material shows the lowest temperature resistance, while it can be observed that the reduction in material mass loss during testing was, on average, 0.074 g with the highest through friction force of 10.4 N, specific wear work *We_f_* with the highest relative deviation. Even though the TT-4 Tribotester test rig is designed to have the least temperature influence on the measured values, it is possible that these variations may be the temperature influence due to frictional heating. The opposite can be more or less observed for the ABS material, which shows the highest average material loss of 0.34 g, with an average friction force of 8 N, which is neither among the highest nor lowest values recorded, but the specific work wear is recorded as the lowest with the lowest relative standard deviation.

In our research, it was found that the friction force of PLA samples is significantly higher than that of PETG, which is in direct contradiction to [50]. This fact may be due to the different scheme and concept of wear measurement, especially the printing conditions. On the contrary, for the research carried out in [51], the trends are consistent. In the case of the comparison between PLA and ABS, similar trends in the magnitude of the frictional force as in the research presented here have been demonstrated. Different trends were observed when comparing PETG and ABS materials.

**Figure 23:**
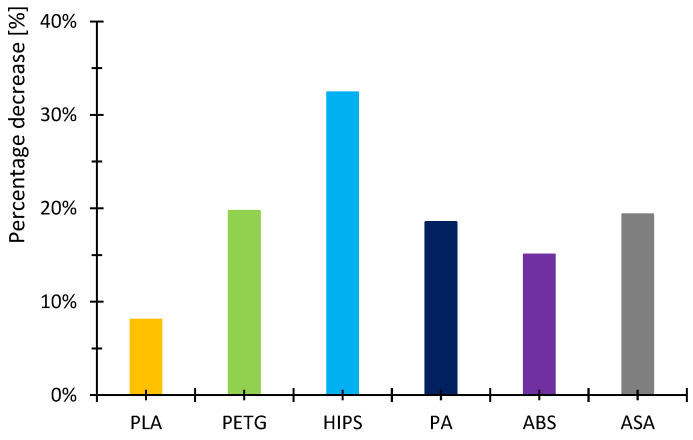
The percentage decrease in storage modulus between 20 °C and 40 °C.

The percentage decrease in storage modulus between 20 °C and 40 °C is shown in the graph in Figure 23. The purpose of this figure is to compare the properties of the different materials. For mechanical designers, it is necessary to know how the material behaves in normal conditions, where, for example, the ambient temperature is not artificially increased above normally acceptable limits. For this reason, a temperature range of 20 °C to 40 °C was selected, and a comparison of the storage modulus or its percentage decrease was made.

In the case of PLA, the storage modulus drops steeply with increasing temperature, indicating a dramatic decrease in the material’s ability to withstand loads. In [52], the researchers test PLA, ABS and PEG material. The samples in this research are produced by injection molding and not by 3D printing as in our example. Anyway, in the case of PLA material, slightly increased storage modulus values can be observed at lower temperatures. The temperature at which a significant decrease occurs is similar. The ABS material did not show such a decrease in storage modulus as the PLA material. In this respect, products made of this material can be used where the material needs to be temperature stable up to approximately 110 °C. In research [52,53], similar trends were measured; however, the magnitude of the storage modulus and, thus, the ability to withstand loads are slightly higher than in other research [52,53]. The PETG material shows a significant decrease in storage modulus from approximately 80 °C. In general, lower values of the storage modulus are demonstrated than in the case of PLA material. Research [25] addresses the effect of layer thickness and frequency in DMA testing. With respect to the layer thickness, which was also used in the present research, identical values of the region where the storage modulus drops dramatically can be observed. The storage modulus magnitude for a well height of 0.3 mm is also consistent with our measured values. This may be due to the fact that the production method used to produce the samples was 3D printing. The storage modulus for the PA material can be observed to drop linearly from approximately 40 °C. The size of the storage module was measured to be smaller in the 3D-printing case than for the PA6 and PA11 materials presented in [54,55]. For the HIPS material, the storage modulus values were measured to be low. The decrease in the modulus and, thus, in the ability to withstand loads decreases linearly, and from about 100 °C, the decrease is significant. Higher values of the storage modulus were measured in [56]. In this study, a decrease in storage modulus around 100 °C was observed, as in our case. Again, the differences between the magnitudes may be due to the different fabrication methodology. Higher storage modulus values were measured for the ASA material, suggesting better load-resisting capabilities than for the ABS material. Less thermal stability is also observed compared to ABS material. Unfortunately, no corresponding studies were found in the investigation that would be useful for discussion.

In terms of technical recommendations, the following can be stated:

- PLA material is characterized by very good print quality, with relatively simple printing parameters. The measured data show low material loss, which corresponds to its higher hardness. Applications with higher operating temperatures should be avoided due to degradation over time and worse ability to resist higher temperatures. It is important to mention the higher storage modulus values and, thus, the greater ability to withstand loads than in the case of, e.g., PETG material.
- PETG material is suitable for prototype production outside of higher-temperature applications. This material can be used in most applications because of its versatility, as it combines the advantages of PLA and ABS materials—ease of printing, higher temperature resistance with slightly lower load capacity due to the storage module compared to, e.g., PLA.
- HIPS cannot be recommended for applications with mechanical stresses because it showed the highest percentage loss of material in relation to its original weight. It exhibits good temperature resistance, but the ability to withstand temperature loads varies quickly.
- PA can be recommended for structural applications. This material has the lowest frictional force, which, combined with good mechanical properties, can be recommended for sliding fits. Its resistance to oils is also an advantage. For applications in sliding fits, it is necessary to consider the magnitude of the normal force with respect to the size of the storage modulus, which decreases relatively rapidly with temperature, which can be a limiting factor in sliding fits applications.
- For structural applications with higher temperatures, ABS can also be used, which, due to its ability to be smoothed with acetone, can, in some cases, positively affect the already quite good friction properties. The ability to withstand the load with respect to the measured values should be acceptable.
- ASA material is characterized by properties that are very similar to ABS material; therefore, its use is also very similar. ASA has the advantage of lower thermal expansion, which makes it much easier to print larger objects, unlike ABS, which has problems printing larger objects. However, ASA has slightly lower temperature resistance than ABS. Measurements showed a slightly higher storage modulus but also a faster decrease with temperature, which may again be a limiting factor in some applications with varying operating temperatures.

## 5. Conclusions

The research presented in this article aims to clarify and help professional and hobby users with the selection of materials for their applications, or to help expand the use of 3D printing in professional practice and, thus, take advantage of the fundamental benefits of this technology. In conclusion, the following can be stated:

- The measurement results show that the glass transition values for the 3D-printing materials tested either agree with or show small deviations from the values reported by the manufacturers. The temperature resistance of the materials varies, with PLA and PA having the lowest temperature resistance, while ASA and ABS have the highest.
- Research has shown that PLA has the lowest temperature resistance and the lowest material weight loss, while ABS has the highest weight loss, but ASA has the highest temperature resistance.
- The damping capacity of materials varies with increasing temperature. PLA shows constant values up to 40 °C, PETG up to 45 °C, PA up to 40 °C, HIPS and ASA up to 60 °C, and ABS up to 40 °C.
- The physical damage of the samples after DMA testing varied between materials. PLA and PETG did not show significant damage, only changes in shape (bent sample, significantly wavy pattern). PA and HIPS showed moderate waviness, while ABS and ASA had low waviness and were planar. HIPS and ASA also showed physical damage at the supports of the three-point bend.

The measured results show that PA material showed the best results in terms of material loss and frictional work. For this reason, it can be concluded that it is more suitable for use in applications with sliding tracks, etc. Conversely, in terms of thermal stress, ASA and ABS materials show better thermal stability, which is certainly an advantage in higher-temperature applications.

Future work should then be directed towards improving these properties, either by developing new materials or by modifying them to improve the dynamic behavior but also by improving the sliding properties by developing coatings whose application to a given surface will improve the friction parameters and reduce wear.

## Figures and Tables

**Table 1:** Printing parameters of samples for wear testing.

Sample Designation	Layer Height [mm]	First Layer Height [mm]	Nozzle Diameter [mm]	Fill [%]	Type of Filling	Printing Temperature [°C]	Filament Color	Brand
PETG-100-030-1;2;3	0.3	0.2	0.4	100	Straightforward	240	White	Filament PM©
PLA-100-030-1;2;3						215	Azure blue	Prusa Research©
ASA-100-030-1;2;3						260	Galaxy Black	Prusa Research©
ABS-100-030-1;2;3						255	White	Prusa Research©
PA-100-030-1;2;3						235	Natural	Filament PM©
HIPS-100-030-1;2;3						220	Grey	C-Tech

**Table 2:** Printing parameters of samples for DMA testing.

Sample Designation	Layer Height [mm]	First Layer Height [mm]	Nozzle Diameter [mm]	Fill [%]	Type of Filling	Printing Temperature [°C]	Filament Color	Brand
PETG-100-030-DMA	0.3	0.2	0.4	100	Straightforward	240	White	Filament PM©
PLA-100-030-DMA						215	Azure blue	Prusa Research©
ASA-100-030-DMA						260	Galaxy Black	Prusa Research©
ABS-100-030-DMA						255	White	Prusa Research©
PA-100-030-DMA						235	Natural	Filament PM©
HIPS-100-030-DMA						220	Grey	C-Tech

**Table 3:** Wear test conditions.

Friction belt parameters	Grain size [µm]	212–180
	Grain material	Silicon carbide
	Carrier [g/m^2^]	paper 270
Test parameters	Sample pressure [kPa]	250 ± 12.5
	Friction belt speed [m/s]	0.2 ± 0.01
	Friction distance [m]	12
	Test time [s]	60
Environmental conditions	Air humidity [%]	40–50
	Temperature [°C]	23 ± 1

**Table 4:** Alicona InfiniteFocus setup for objective magnification 10×.

Numerical aperture [-]	0.3
Working distance [mm]	17.5
Lateral measurement range (X, Y) [mm]	1.62
Lateral measurement range (X × Y) [mm^2^]	2.62
Measurement point distance [μm]	0.88
Measurement noise [nm]	30
Vertical resolution [nm]	100
Vertical measurement range [mm]	16.5
Min. measurable roughness (Ra) [μm]	0.3
Min. measurable radius [μm]	5

**Table 5:** S-parameters and V-parameters of friction belt surface.

S-parameters	Average height of selected area [μm]	41.7
	Root-Mean-Square height of selected area [μm]	53.8
	Maximum peak height of selected area [μm]	266.3
	Maximum valley depth of selected area [μm]	316.3
	Maximum height of selected area [μm]	582.5
	Skewness of selected area [-]	0.827
	Kurtosis of selected area [-]	4.1
V-parameters	Core roughness depth, Height of the core material [μm]	125.6
	Reduced peak height, mean height of the peaks above the core material [μm]	80.9
	Reduced valley height, mean depth of the valleys below the core material [μm]	30.4
	The fraction of the surface which consists of peaks above the core material [%]	13.9
	The fraction of the surface which will carry the load [%]	93
	Peak material volume of the topographic surface [mL/m^2^]	4.04
	Core material volume of the topographic surface [mL/m^2^]	44.8
	Core void volume of the surface [mL/m^2^]	69.7
	Valley void volume of the surface [mL/m^2^]	4.2

**Table 6:** DMA testing conditions.

Sample No.	Initial Temperature [°C]	Heating Rate [°C/min]	Maximum Test Temperature [°C]	Load Frequency [Hz]	Max. Force Amplitude [N]
PA_100_030_DMA_BEND-1	23	2	145	3	40
ABS_100_030_DMA_BEND-1			115		
PLA_100_030_DMA_BEND-1			90		
ASA_100_030_DMA_BEND-1			130		
HIPS_100_030_DMA_BEND-1			130		
PETG_100_030_DMA_BEND-1			120		

**Table 7:** Brief summary of results from measurements on the wear measurement device (green color—best result, red color—worst result, orange color—intermediate result).

Material	Loss of Weight	Friction Force	Friction Work
PLA_100_030	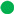	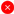	
PETG_100_030	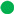	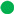	
HIPS_100_030	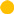	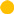	
PA_100_030	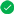	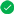	
ABS_100_030	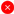	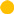	
ASA_100_030	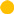	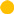	

**Table 8:** Specific wear work *e_f_* [J/g].

Sample No.	Sample Designation/Material					
	PLA-100-030	PETG-100-030	HIPS-100-030	PA-100-030	ABS-100-030	ASA-100-030
1	1775.3	1020.8	571.7	1908.9	227	389.0
2	1987.5	1076.2	529.7	1886.3	329.4	578.2
3	1787.9	1382.5	601.6	2370.2	345.6	577.6
4	1221.7	1452.8	479.7	1946.7	274.0	
5	3316.5 *	959.1	392.5	1710.2	263.7	
6	1197.9	916.9	424.8	1417.4	275.6	
Average	1881.1 * 1594	1134.7	500	1873.3	285.9	514.9
Standard deviation	773.7 * 360.8	226.8	82.4	312.5	44.0	109.1
Relative standard deviation	41% * 23%	20%	16%	17%	15%	21%

Based on Chauvenet’s criterion for *n* = 6, T = 1.732, the fifth sample value PLA-100-030 was removed. The results after removal are marked with “*”.

**Table 9:** Measured values in the range (20–40) °C.

Material	*E*′	*E*″	*tan*(*δ*)
PLA	1980–1820	~60	~0.03
PETG	1100–883	20–22	~0.02
HIPS	762–515	37.8–42.7	0.05–0.08
PA	891–726	43.1–55.7	0.05–0.075
ABS	1030–875	56.7–56.1	0.055–0.065
ASA	1210–976	36.1–34.3	~0.03

**Table 10:** Deformation characteristics of individual samples.

Sample No.	Character of Deformation	
	Front View	Side View
PLA_100_030_DMA_BEND-1	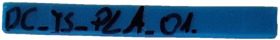	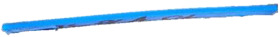
PETG_100_030_DMA_BEND-1	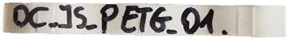	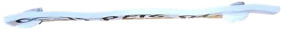
HIPS_100_030_DMA_BEND-1	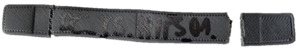	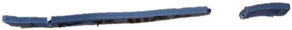
PA_100_030_DMA_BEND-1	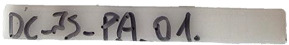	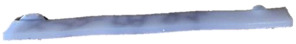
ABS_100_030_DMA_BEND-1	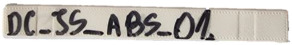	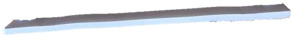
ASA_100_030_DMA_BEND-1	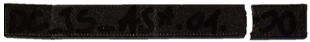	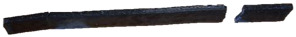

**Table 11:** Recapitulation of measured temperatures and comparison with the available literature.

Material	Glass Transition Temperatures *T_g_* [°C]			
	*T*_*g*δ_	*T*_*gE*″_	*T*_*gE*′_	Table Value for 3D Printing Filaments Others
PLA	64.3	59.7	59.1	55–65 [36,37,38,39,40,41,42]
PETG	87.2	80.8	80.7	75–80–85 [37,39,42,43]
HIPS	114	89.4	-	100–110 [37,44]
PA	90.6	49.7	44.4	55 [43,45,46]
ABS	≥115	112	110.8	105–110 [38,42,43,47,48]
ASA	116	107	105.1	108–115 [40,43,49]

## Data Availability

Data are contained within the article.
